# The sparse macroecology of microbiology

**DOI:** 10.1073/pnas.2318518120

**Published:** 2024-01-22

**Authors:** Christopher P. Kempes

**Affiliations:** ^a^The Santa Fe Institute, Santa Fe, NM 87501

Ecology is often defined in terms of the interactions among species. Macroecology aims to take a global view of the general patterns of all ecological processes including the interactions among organisms. This high-level perspective has created many challenges for ecology ranging from how to identify all of the interactions in the first place to surprising results by Robert May in the 1970s that most large complex systems defined by interaction matrices are unstable (1).

It becomes even more challenging to understand ecological systems if one considers higher-order interactions, where, for example, the interaction of any two species depends on the presence or absence of a third species. In such contexts, there are even more types of interactions to count and the dynamics would seemingly be even more unwieldy. Understanding all of the interactions among microorganisms is a daunting task for two reasons: First is the curse of combinatorics. Imagine that you have S species; this allows for $2^{S}$ presence–absence community compositions. If S is just 10, a modest microbial community, then $2^{6}$ = 1,024 (2). Do we need to observe all of these to infer the full set of species interactions? Another challenge is the sheer number of species that can exist in microbial ecosystems. That is, S can be quite large. These problems clearly indicate the need for new tools. Furthermore, how should we approach this problem when we often only have limited observations of a system? These are all of the challenges and questions addressed in the new paper by Arya et al. (2).

The authors use compressive sensing, which is a tool from signal processing aimed at reconstructing a signal from a few measurements. The challenge for both signal processing and ecology is that one is often trying to find solutions to a small number of equations for contexts with a large number of free variables. However, this is possible under the condition of sparsity, that is, if most of the coefficients of the equations are close to zero. In an ecological context, this would imply that most of the values in an interaction matrix are zero. The authors take this starting place for compressive sensing and assume sparsity and then they fit the best sparse representation.

The authors produce a tool that creates a map from initial to final species compositions. This map works for any initial composition even if produced on a small amount of data. Said another way, once the model is fit, it is possible to predict the steady-state ecology given any starting community. To validate the method, the authors first test the approach on synthetic ecological data, where they know the true answer. They find that sampling only 10% of the full data provides reasonable predictions. They then show that the method works well for diverse microbial ecosystems ranging from soil to the fly and human gut.

The big surprise in their paper is that this method works so well for predicting microbial ecology. The fact that the best-fit sparsity representation has predictive power in observed systems tells us that the interaction matrix is sparse. In fact, the authors find that both pairwise and higher-order interactions are sparse.

It is important to note that prediction is not perfect for observed data. The $R^{2}$ between prediction and observation can be around 0.80, but the model does provide an impressive starting point given very limited data. This starting point is useful for employing limited data to make predictions, design future experimentation or data collection, and to build and test theories in a specific context.

Another key feature of the compressive sensing approach is that it outperforms much more complicated and opaque machine learning perspectives. Many people are happy to call even the simplest linear regression for prediction “machine learning (ML),” and so the key consideration for ML really becomes about how complicated the fitting algorithm is and how much it predicts. Compressive sensing is a straightforward regression procedure because of the simplifying assumptions of sparsity, and the authors show that it outperforms two other ML procedures that are commonly used and are slightly more complicated.

Stepping back, this work is part of a growing body of theoretical and empirical work uncovering the structure and implications of higher-order interactions in ecology. For example, recent work has shown that higher-order interactions stabilize dynamics (3) and lead to a greater number of feasible coexistences (4). More broadly, this work is also part of the exciting emergence of a macroecology of microbial systems. There are several theories of macroecology that have been developed over the last century which show law-like regularities across diverse ecological systems (5). These include, to name a few, scaling relationships between various organism traits, species abundance distributions, and the demographics of body size in an ecosystem. In recent years, each of these regularities and/or theories has been mapped onto the microbial world (e.g., refs. 6–10). In addition, recently there has been a push to begin to understand the synthesis between these theories (5). The paper by Arya et al. adds an exciting new tool for uncovering the effective interactions of species and showing that these are sparse which will allow certain theories to be compared with data and refined. Looking forward, an open challenge is to connect automatic or machine learning models to analytic theory. This is a general challenge for the emerging ecology of big data and is meant as an open call to the community rather than a critique. Importantly, compressive sensing offers a much simpler and transparent ML approach than most others, and sparsity gives us hope for simple theories.

“The technique developed by Arya et al. provides an exciting new tool for uncovering interactions in ecological systems.”

The roadmap is then to use this method as a way to reveal the interactions within a system and then to build, compare, validate, and update theories based on those results. In combination, these perspectives and theories provide many avenues for a better understanding and prediction of microbial ecosystems ranging from those in the human gut, to industrial scenarios like food production and waste treatment, to forecasting the future ecology of soils, lakes, and oceans. Such theories will allow us to better understand the climate system as well as our own health. The technique developed by Arya et al. provides an exciting new tool for uncovering interactions in ecological systems.
